# Alcohol Consumption and Dietary Patterns: The FinDrink Study

**DOI:** 10.1371/journal.pone.0038607

**Published:** 2012-06-12

**Authors:** Timothy O. Fawehinmi, Jenni Ilomäki, Sari Voutilainen, Jussi Kauhanen

**Affiliations:** 1 Institute of Public Health and Clinical Nutrition, University of Eastern Finland, Kuopio, Finland; 2 School of Pharmacy and Medical Sciences, Quality Use of Medicines and Pharmacy Research Centre, Sansom Institute, University of South Australia, Adelaide, Australia; University of Bath, United States of America

## Abstract

The aim of this population-based study was to investigate differences in dietary patterns in relation to the level of alcohol consumption among Finnish adults. This study was part of the FinDrink project, an epidemiologic study on alcohol use among Finnish population. It utilized data from the Kuopio Ischaemic Heart Disease Risk Factor Study. A total of 1720 subjects comprising of 816 men and 904 women aged 53–73 years were included in the study in 1998–2001. Food intake was collected via a 4-day food diary method. Self-reported alcohol consumption was assessed with quantity-frequency method based on the Nordic Alcohol Consumption Inventory. Weekly alcohol consumption was categorized into three groups: non-drinkers (<12 grams), moderate drinkers (12–167.9 grams for men, 12–83.9 grams for women) and heavy drinkers (≥168 grams for men, ≥84 grams for women). Data were analyzed for men and women separately using multiple linear regression models, adjusted for age, occupational status, marital status, smoking, body mass index and leisure time physical activity. In women, moderate/heavy drinkers had lower fibre intake and moderate drinkers had higher vitamin D intake than non-drinkers. Male heavy drinkers had lower fibre, retinol, calcium and iron intake, and moderate/heavy drinkers had higher vitamin D intake than non-drinkers. Fish intake was higher among women moderate drinkers and men moderate/heavy drinkers than non-drinkers. In men, moderate drinkers had lower fruit intake and heavy drinkers had lower milk intake than non-drinkers. Moderate drinkers had higher energy intake from total fats and monosaturated fatty acids than non-drinkers. In contrast, energy intake from carbohydrates was lower among moderate/heavy drinkers than non-drinkers. In conclusion, especially male heavy drinkers had less favorable nutritional intake than moderate and non-drinkers. Further studies on the relationship between alcohol consumption and dietary habits are needed to plan a comprehensive dietary intervention programs in future.

## Introduction

Ethanol can replace almost 60 percent of daily calorie intake of adult chronic drinkers in some cases leading to malnutrition. Excessive consumption of alcohol may result in nutrient deficiencies reflecting variability in their clinical significance. Alcohol consumption changes metabolism of most nutrients and the consequences of these distortions may play a significant role in the pathogenesis of liver disease. Heavy alcohol consumption can alter carbohydrate and fibre metabolism causing alcoholic hypoglycemia and loss of protein. Alcohol intake inhibits the breakdown of triglycerides thus reducing the free fatty acid levels in the body [1]. The calorie-wasting effect of alcohol usually makes alcohol consumers to have altered nutritional status coupled with effect of rough lifestyle. This is partly as a result of nutritional distortion arising from associated illness like cancer, chronic liver diseases or infections [2], [3].

A study assessing the nutritional status of 77 alcoholic patients in Spain, consuming more than 150 grams/day for longer than 5 years, found that the lean mass was decreased in alcohol drinkers which is related to the liver function derangement and alcohol consumption, and is also linked to increased mortality [3]. Dietary patterns are important screening instruments to prevent non-healthy eating habits such as consuming high-salt and low-fibre diet in alcohol drinkers. Diets that are in line with the present recommended nutritional guidelines have been shown to decrease the several risk factors for cardiovascular diseases [4].

Studies have been carried out in the context of alcohol drinking and nutritional patterns in several countries to assess the confounding roles of specific nutrients, dietary habits and beverage preferences [5]–[8] but there is a paucity of research on the association of alcohol consumption and dietary patterns. As part of the FinDrink study [9], this study is expected to provide information on the differences in the eating habits in relation to level of alcohol consumption in a Finnish population sample.

## Methods

### Ethics Statement

The Kuopio Ischaemic Heart Disease Risk Factor (KIHD) Study has been approved by the Research Ethics Committee of the University of Eastern Finland, Kuopio Campus. All the participants signed a written informed consent.

### Study Population

The data was obtained from the Kuopio Ischaemic Heart Disease Risk Factor (KIHD) Study. KIHD started as a population based cohort study in 1984 aiming at investigating the risk factors for chronic illnesses such as cardiovascular diseases amongst middle-age Finnish men living in eastern part of Finland [9]. Subjects were a random sample of 2682 men living in the Kuopio city and the neighboring rural communities, age stratified into four levels: 42, 48, 54 and 60 years at the baseline examination. Eligible participants must had been alive and reside in the sampling catchment area during the period of the study [10]. The subjects were recruited in two cohorts. The first cohort included 1166 men who received their baseline examination between March 1984 and August 1986. The second cohort consisted of 1516 men and they had their baseline examination between August 1986 and December 1989. While the first cohort was examined only at baseline, the second cohort was invited for re-examinations 11 years later in 1998–2001 to which 854 out of 1516 participated. The reasons for lost of follow-up from baseline to the 11-year examination were lack of critical baseline data that determined the eligibility to the follow-up examinations (n = 287), death (n = 136), refusal (n = 132), severe illness (n = 50), no contact (n = 44), no address (n = 3) and other (n = 6). In 1998–2001, a random sample of 1351 women in the equivalent age group was invited from which 920 participated. The reasons for non-participation among women were refusal (n = 168), severe illness (n = 97), pre-menopause (n = 54), no contact information (n = 51), death (n = 14), migration (n = 10), no address (n = 3) and others (n = 34).

The present study utilized the data from the examinations conducted in 1998–2001 including 920 women and 854 men, aged 53–73 years. After excluding those with missing data on alcohol consumption and dietary intake, 904 women and 816 men were analyzed.

### Assessment of Alcohol Consumption

The level of alcohol consumption was evaluated through the use of self-administered questionnaire. Self reported alcohol consumption was assessed via quantity-frequency method based on the Nordic Alcohol Consumption Inventory [11] from which the average weekly alcohol consumption was calculated. These questions comprised of the quantity and frequency of alcohol consumption during the preceding year. One unit of alcohol contains an average 12 grams of 100% ethanol; (light beer = 11 grams, glass of wine = 12 grams and shot of hard liquor = 14 grams).

Men consuming at least 14 units of alcohol per week (≥168 grams) were defined as heavy drinkers, those consuming between 1–13.9 units (12–167.9 grams) of alcohol weekly constitute the moderate drinkers while weekly alcohol intake of <1 unit (11.9 grams) represent non-drinker group. Among women, heavy drinkers were defined as consumption of ≥7 units/week (≥84 grams) alcohol while moderate drinkers were defined as consuming 1–6.9 units/week (12–83.9 grams). Non-drinkers were defined as consuming <1 unit/week (<12 grams). The definition of heavy drinking is in accordance with the National Institute of Alcohol Abuse and Alcoholism [12] and Dietary Guidelines for Americans 2010 [13]. We decided to use lower levels than recommended by World Health Organization (low-risk drinking ≤20 grams/day for women and ≤40 grams/day for men) [14], because half of our sample was 65 years or older. The recommendations for alcohol consumption for people aged 65 years or older are lower [12].

### Assessment of Food Consumption

The nutritional consumption patterns were assessed using a 4-day food record diary [15]. The nutrient and energy intake was estimated through validated Nutrica software version 2.5 which employs Finnish values of nutrient food composition and takes into account losses from vitamins during food preparation [16]. The Nutrica software was developed by the Research Center of the Social Insurance Institution of Finland and it contains a broad database of over 1300 food items and more than 30 nutrient groups. Instructions were given to the participants by the nutritionist on how to fill in the food diary. These instructions included estimating portion sizes, the type of food, the time of day each participant ate or drank and the place of eating. The participants were given a food record sample before filling in the diary. Completed food records were checked by a nutritionist. The participants were advised not to change their normal eating habits.

The food intake groups include whole grain products such as rye products, fruits, berries, vegetables independently, total amount of fruits and vegetables, potatoes, root vegetables, milk, sour milk products, cheese, beef, fish, coffee and tea. The composition of whole grain products embraced different breads, flakes, bran, germ and muesli products, not including refined flour products. The rye product comprised of different rye breads, rye flour, flakes, bran and malt. The vegetable group contains all fresh and frozen vegetables, apart from pickled and canned vegetables. Carrots, Swedish turnip (swede), turnip and beetroot are types of the roots, which are frequently consume in Finland where included with the exception potatoes, which were assigned into a separate group. The constituent of fruits group are fresh, canned and dried fruits as well as fruit nectars, while other juices were added into the fruit juices category. The berries group included fresh and frozen berries, crushed lingonberries and lingonberry jam which are commonly manufactured in Finland and they contain no sugar. The fish total group comprises of all fish and fish product. Milk category included fresh milk and whole milk. Sour milk category also contains yoghurt.

### Demographic Factors

Age and education were measured in years. Marital status was defined as married/couple, divorced/widow or single. Occupation types were differentiated into farmer, blue collar jobs, and white collars jobs while place of dwelling was either living in Kuopio city or rural areas. Demographic factors were collected via self-administered questionnaire.

### Behavioral and Biological Risk Factors

Diastolic and systolic blood pressure were measured using a random–zero mercury sphygmomanometer. Blood pressure was estimated by taking mean of six measurements which are three in supine, one in standing and two in sitting position [17]. The energy expenditure by conditioning leisure time physical activities were assessed by using a 12-month history modified from the Minnesota Leisure Time Physical Activity Questionnaire. The intensity of physical activity was measured in metabolic units (metabolic equivalent of task MET or metabolic equivalent of oxygen uptake) and one MET is equivalent to an energy expenditure of approximately 1 kcal/kg * hour and oxygen consumption of 3.5 ml/kg * minute. Energy expenditure expressed in kcal/week for every activity was estimated by multiplying the metabolic index of the activity (MET * hour/week) by the weight of the body in kilograms [18].

Body mass index (BMI) was measured as weight in kilograms divided by the square of the height in meters. Low density lipoprotein (LDL) and high density lipoprotein (HDL) portions were extracted from fresh serum by a method of combine ultracentrifugation and precipitation during the medical examination [17]. Smoking status and cigarette smoking were collected using self-administered questionnaire.

### Statistical Analysis

Descriptive statistics are presented as means (± standard deviation, SD) and proportions. For baseline characteristics, categorical variables were analyzed using Pearson’s chi square test and continuous variables using one-way analysis of variance (ANOVA) and Kruskal Wallis Test. Multivariate models for men and women separately were computed using multiple linear regression adjusted with age, occupational status, marital status, smoking, body mass index and leisure time physical activity. We calculated adjusted beta coefficients (β) with 95% confidence intervals (CI) and P values <0.05 were considered statistically significant. Non-drinkers were used as the reference group in all analyses. When assumptions of dependent variable were not met, we transformed the variable either taking a logarithm (log10) or a square root of the value. Statistical Package for the Social Sciences (SPSS) for windows version 17.0 was used to compute baseline characteristics and SAS version 9.3 was used to compute multivariate analyses.

## Results

### Demographic Factors

Among both sexes, alcohol consumption was related to the type of occupation and years of education (P<0.001) (Table 1). In women, 87.5% of heavy drinkers were white collar workers while 79.4% and 61.9% represent white collar workers among moderate and non-drinkers, respectively. Among men, 60% of heavy drinkers were white collar workers. Among moderate and non-drinkers the proportions of white collar workers were 48.9% and 35.7%, correspondingly.

**Table 1:** Baseline characteristics of participants according to alcoholic consumption.

	Alcohol consumption (g/wk)							
	Women				Men			
	<12.0 (none)	12.0–83.9 (moderate)	≥84 (heavy)		<12.0 (none)	12.0–167.9 (moderate)	≥168.0 (heavy)	
	n = 604	n = 252	n = 48	p-value*	n = 252	n = 444	n = 120	p-value*
Age, years	64.0±6.5	61.8±6.1	59.0±5.9	<0.001	64.5±6.1	61.9±6.3	60.3±6.6	<0.001
Place of dwelling				0.074				0.037
Kuopio city, %(n)	58.6(354)	61.5(155)	75.0(36)		48.0(121)	39.6(176)	35.8(43)	
Occupation				<0.001				<0.001
Farmer, %(n)	19.0(115)	7.5(19)	2.1(1)		16.7(42)	9.0(40)	6.7(8)	
Blue collar, %(n)	19.0(115)	13.1(33)	10.4(5)		47.6(120)	42.1(187)	33.3(40)	
White collar, %(n)	61.9(374)	79.4(200)	87.5(42)		35.7(90)	48.9(217)	60.0(72)	
Marital status				0.082				0.001
Married/Couple, %(n)	64.9(392)	65.9(166)	77.1(37)		82.1(207)	87.2(387)	85.2(695)	
Single, %(n)	10.1(61)	5.6(14)	4.2(2)		9.9(25)	3.2(14)	2.5(3)	
Divorced/Widow, %(n)	25.0(151)	28.6(72)	18.8(9)		7.9(20)	9.7(43)	13.3(16)	
Education, years	9.3±3.3	10.3±3.5	10.8±3.4	<0.001	8.6±3.1	9.8±3.7	10.3±3.7	<0.001
Cigarettes, day	0.5±2.4	1.4±4.4	3.8±7.4	<0.001	0.5±2.4	1.7±4.9	5.3±7.4	<0.001
Smoking status				<0.001				<0.001
Non-smoker, %(n)	86.8(524)	68.7(173)	47.9(23)		46.4(117)	36.0(160)	21.70(26)	
Previous smoker, %(n)	8.3(50)	18.7(47)	27.1(13)		43.3(109)	45.0(200)	46.7(56)	
Current smoker, %(n)	5.0(30)	12.7(32)	25.0(12)		10.3 (26)	18.9(84)	31.7(38)	
Energy expenditure, Kcal/d	175.5±178.2	219.8±272.6	123.2±166.2	0.002	188.2±202.6	190.7±195.0	147.6±145.0	0.059
Body mass index, kg/m^2^	28.5±5.3	27.6±4.5	29.4±5.3	0.046	27.1±3.6	27.3±3.5	28.1±4.1	0.110
Mean systolic BP, mmHg	138.0±17.6	135.3±16.8	139.4±15.2	0.090	134.1±18.3	133.7±16.7	136.7±15.7	0.230
Mean diastolic BP, mmHg	80.0±8.6	81.0±8.5	83.6±7.7	0.009	80.3±9.6	82.0±8.9	84.9±9.2	0.000
S-total cholesterol, mmol/l	5.7±0.9	5.8±0.9	5.7±0.8	0.250	5.2±0.9	5.3±1.0	5.3±0.9	0.012
S-LDL-Chol, mmol/l	3.7±0.9	3.7±0.9	3.7±0.8	0.949	3.4±0.8	3.5±1.0	3.4±0.9	0.164
S-HDL-Chol, mmol/l	1.3±0.3	1.4±0.3	1.4±0.3	<0.001	1.1±0.3	1.1±0.3	1.2±0.3	<0.001

Abbreviations: BP = Blood pressure; S-LDL-Chol = Serum low density lipoprotein cholesterol; S-HDL-Chol = Serum High density lipoprotein cholesterol.

* Calculated using Pearson’s chi-square test for categorical and ANOVA or Kruskal-Wallis test for continuous variables. Data expressed as the mean ± standard deviation unless otherwise indicated.

### Behavioral and Biological Risk Factors

The mean energy expenditure by conditioning leisure time physical activity was lower among the heavy drinkers compared with moderate and non-drinkers in women (P = 0.002) (Table 1). Heavy drinkers had slightly higher levels of HDL-cholesterol compared to non-drinkers (P<0.001 for both sexes). The prevalence of cigarette smoking was also significantly higher amongst heavy drinkers than moderate and non-drinkers (P<0.001). Of male heavy drinkers, 31.7% were current smokers while of moderate and non-drinkers 18.9% and 10.3% respectively were current smokers.

### Alcohol Consumption and Nutrient Intake

In women, moderate (β = −1.24, P = 0.016) and heavy (β = −2.88, P = 0.005) drinkers had lower fibre intake compared to non-drinkers after adjusting for all covariates (Table 2). Moderate drinkers also had slightly higher intake of vitamin D (β = 0.12, P = 0.011) than non-drinkers. In men, there was a lower intake of fibre (β = −4.09, P<0.001), retinol (β = −0.19, P = 0.035), calcium (β = −0.12, P = 0.018) and iron (β = −1.19, P = 0.019) among heavy drinkers compared to non-drinkers. In contrast, compared to non-drinkers, moderate (β = 0.19, P<0.001) and heavy (β = 0.18, P = 0.016) drinkers had higher intake of vitamin D.

**Table 2:** Nutrient intake based on the 4-Day Food Diary according to alcohol consumption.

	Women					Men				
	Alcohol category	mean±SD*	β**	95% CI	P-value	Alcohol category	mean±SD*	β**	95% CI	P-value
Fibre, g	None (n = 604)	20.2±6.8	ref			None (n = 252)	26.4±9.6	ref		
	Moderate (n = 252)	19.1±6.3	−1.24	−2.25, −0.23	0.016	Moderate (n = 444)	24.8±9.9	−1.49	−3.03, 0.05	0.057
	Heavy (n = 48)	17.3±6.2	−2.88	−4.90, −0.86	0.005	Heavy (n = 120)	21.2±9.2	−4.09	−6.30, −1.87	<0.001
Retinol equivalent, µg	None (n = 604)	1127±1951	ref			None (n = 252)	1140±1571	ref		
	Moderate (n = 252)	1211±2006	0.03	−0.08, 0.15	0.587	Moderate (n = 444)	1393±2379	0.03	−0.10, 0.15	0.677
	Heavy (n = 48)	1224±1350	0.01	−0.22, 0.24	0.923	Heavy (n = 120)	1052±1654	−0.19	−0.37, −0.01	0.035
Vitamin D, µg	None (n = 604)	5.1±4.1	ref			None (n = 252)	6.8±6.4	ref		
	Moderate (n = 252)	5.7±4.6	0.12	0.03, 0.21	0.011	Moderate (n = 444)	8.4±7.7	0.19	0.08, 0.29	<0.001
	Heavy (n = 48)	4.9±3.7	0.05	−0.14, 0.23	0.622	Heavy (n = 120)	8.0±7.6	0.18	0.03, 0.33	0.016
Calcium, mg	None (n = 604)	908±350	ref			None (n = 252)	1136±451	ref		
	Moderate (n = 252)	934±344	0.01	−0.05, 0.07	0.694	Moderate (n = 444)	1113±472	−0.01	−0.08, 0.06	0.696
	Heavy (n = 48)	882±343	−0.09	−0.22, 0.03	0.141	Heavy (n = 120)	1000±449	−0.12	−0.22, −0.02	0.018
Iron, mg	None (n = 604)	9.8±3.5	ref			None (n = 252)	12.7±4.3	ref		
	Moderate (n = 252)	10.0±3.6	0.27	−0.28, 0.81	0.341	Moderate (n = 444)	12.9±4.4	0.07	−0.63, 0.76	0.852
	Heavy (n = 48)	10.4±3.9	0.50	−0.60, 1.60	0.371	Heavy (n = 120)	11.5±3.8	−1.19	−2.18, −0.19	0.019
Folate, µg	None (n = 604)	217±73	ref			None (n = 252)	252±82	ref		
	Moderate (n = 252)	225±71	0.02	−0.03, 0.07	0.407	Moderate (n = 444)	256±79	0.01	−0.04, 0.06	0.673
	Heavy (n = 48)	210±59	−0.05	−0.14, 0.05	0.310	Heavy (n = 120)	236±79	−0.05	−0.12, 0.03	0.211
Vitamin C, mg	None (n = 604)	89.7±57.0	ref			None (n = 252)	82.0±61.1	ref		
	Moderate (n = 252)	92.0±59.0	−0.01	−0.11, 0.08	0.760	Moderate (n = 444)	79.4±57.9	−0.06	−0.17, 0.05	0.272
	Heavy (n = 48)	98.7±64.8	0.06	−0.13, 0.25	0.559	Heavy (n = 120)	77.1±55.1	−0.08	−0.23, 0.08	0.337

* unadjusted means ± standard deviation.

** beta coefficient adjusted with age, occupation, marital status, smoking, body mass index and leisure time physical activity.

### Alcohol Consumption and Food Intake

Women who were moderate drinkers had higher fish intake compared to non-drinkers (β = 0.83, P = 0.035) (Table 3). Among men, fish intake was higher among moderate (β = 1.40, P = 0.004) and heavy (β = 1.91, P = 0.007) drinkers than among non-drinkers. Furthermore, fruit intake was lower among moderate drinkers (β = −1.28, P = 0.020) and milk intake among heavy drinkers (β = −0.49, P<0.001) compared to non-drinkers.

**Table 3:** Daily food intake based on the 4-Day Food Dairy according to alcohol consumption.

	Women					Men				
	Alcohol category	mean±SD*	β**	95% CI	P-value	Alcohol category	mean±SD*	β**	95% CI	P-value
Vegetables, g	None (n = 604)	151.7±185.2	ref			None (n = 252)	126.3±154.6	ref		
	Moderate (n = 252)	179.7±216.8	0.11	−0.05, 0.27	0.189	Moderate (n = 444)	137.0±168.0	−0.02	−0.20, 0.16	0.832
	Heavy (n = 48)	171.8±175.4	0.23	−0.10, 0.56	0.169	Heavy (n = 120)	166.5±241.6	0.11	−0.16, 0.37	0.420
Fruits, g	None (n = 604)	161.7±216.2	ref			None (n = 252)	161.7±216.2	ref		
	Moderate (n = 252)	177.8±261.6	−0.36	−1.43, 0.71	0.504	Moderate (n = 444)	181.4±260.0	−1.28	−2.37, −0.20	0.020
	Heavy (n = 48)	191.0±234.3	−0.26	−2.41, 1.89	0.813	Heavy (n = 120)	135.8±171.8	−1.23	−2.79, 0.33	0.122
Legumes, g	None (n = 604)	4.8±17.6	ref			None (n = 252)	4.9±13.1	ref		
	Moderate (n = 252)	5.6±13.1	0.14	−0.10, 0.38	0.247	Moderate (n = 444)	4.2±12.3	−0.06	−0.30, 0.17	0.593
	Heavy (n = 48)	5.4±17.4	0.00	−0.47, 0.48	0.996	Heavy (n = 120)	4.6±10.9	0.03	−0.31, 0.36	0.877
Fish total, g	None (n = 604)	62.2±105.9	ref			None (n = 252)	79.4±122.6	ref		
	Moderate (n = 252)	68.6±97.4	0.83	0.06, 1.60	0.035	Moderate (n = 444)	100.5±151.3	1.40	0.44, 2.36	0.004
	Heavy (n = 48)	70.0±88.7	1.35	−0.19, 2.90	0.086	Heavy (n = 120)	105.2±160.6	1.91	0.52, 3.29	0.007
Beef g	None (n = 604)	71.0±109.2	ref			None (n = 252)	82.4±109.8	ref		
	Moderate (n = 252)	71.8±82.5	0.21	−0.48, 0.90	0.555	Moderate (n = 444)	91.4±106.3	0.08	−0.69, 0.86	0.831
	Heavy (n = 48)	92.0±103.3	0.95	−0.45, 2.34	0.183	Heavy (n = 120)	117.4±148.1	0.68	−0.44, 1.81	0.231
Milk, g	None (n = 604)	422.1±529.2	ref			None (n = 252)	664.1±817.1	ref		
	Moderate (n = 252)	358.4±471.3	−0.10	−0.28, 0.08	0.267	Moderate (n = 444)	497.3±458.3	−0.13	−0.33, 0.07	0.213
	Heavy (n = 48)	285.0±326.4	−0.33	−0.69, 0.03	0.077	Heavy (n = 120)	393.3±446.3	−0.49	−0.78, −0.20	<0.001
Sour milkproducts, g	None (n = 604)	211.6±350.7	ref			None (n = 252)	208.5±347.7	Ref		
	Moderate (n = 252)	219.2±373.0	0.19	−1.23, 1.61	0.790	Moderate (n = 444)	194.8±381.8	−0.72	−2.34, 0.90	0.382
	Heavy (n = 48)	143.6±184.8	−1.71	−4.56, 1.14	0.240	Heavy (n = 120)	185.5±467.8	−1.35	−3.68, 0.98	0.256
Butter, g	None (n = 604)	16.5±28.3	ref			None (n = 252)	29.0±42.4	Ref		
	Moderate (n = 252)	16.0±27.7	−0.00	−0.37, 0.37	0.996	Moderate (n = 444)	26.4±44.8	−0.04	−0.54, 0.46	0.876
	Heavy (n = 48)	16.8±24.4	0.01	−0.73, 0.76	0.973	Heavy (n = 120)	24.6±35.7	−0.10	−0.81, 0.61	0.783
Coffee, g	None (n = 604)	546.0±608.5	ref			None (n = 252)	592.2±651.0	ref		
	Moderate (n = 252)	552.7±553.8	0.33	−1.25, 1.90	0.684	Moderate (n = 444)	641.0±671.8	1.04	−0.68, 2.77	0.235
	Heavy (n = 48)	568.5±788.4	−1.27	−4.44, 1.89	0.429	Heavy (n = 120)	597.3±584.6	−0.27	−2.76, 2.21	0.830
Whole grain, g	None (n = 604)	154.1±142.4	ref			None (n = 252)	236.6±198.1	ref		
	Moderate (n = 252)	141.0±171.1	−0.11	−0.23. 0.01	0.073	Moderate (n = 444)	228.4±220.6	−0.07	−0.19, 0.06	0.323
	Heavy (n = 48)	140.3±124.0	−0.05	−0.28, 0.19	0.699	Heavy (n = 120)	201.2±199.3	−0.09	−0.28, 0.09	0.321
Tea, g	None (n = 604)	151.5±361.2	ref			None (n = 252)	139.6±236.6	ref		
	Moderate (n = 252)	141.3±345.7	−0.23	−1.67, 1.21	0.752	Moderate (n = 444)	152.2±335.9	0.54	−1.00, 2.04	0.484
	Heavy (n = 48)	153.4±359.9	0.64	−2.26, 3.53	0.665	Heavy (n = 120)	161.4±372.3	1.33	−0.83, 3.49	0.228
Rye products, g	None (n = 604)	102.1±108.1	ref			None (n = 252)	173.1±162.9	ref		
	Moderate (n = 252)	92.2±108.0	−0.55	−1.21, 0.11	0.105	Moderate (n = 444)	160.9±181.3	−0.76	−1.70, 0.18	0.111
	Heavy (n = 48)	89.9±110.9	−0.84	−2.18, 0.49	0.215	Heavy (n = 120)	157.8±204.7	−0.47	−1.82, 0.88	0.498

* unadjusted means ± standard deviation.

** beta coefficient adjusted with age, occupation, marital status, smoking, body mass index and leisure time physical activity.

### Alcohol Consumption and Energy Intake

Moderate drinkers had higher energy intake from total fats and monosaturated fatty acids than non-drinkers (women β = 1.24, P = 0.006 and β = 0.56, P = 0.003; men β = 1.05, P = 0.030 and β = 0.42, P = 0.032, respectively) (Table 4). Women who were moderate drinkers had higher intake of polysaturated fatty acids as well (β = 0.30, P = 0.006). In contrast, energy intake from carbohydrates was lower among moderate and heavy drinkers than non-drinkers among men (β = −3.49, P<0.001 for moderate drinkers and β = −7.56, P<0.001 for heavy drinkers) and women (β = −3.48, P<0.001 for moderate drinkers, β = −6.47, P<0.001 for heavy drinkers). Total energy intake did not differ according to drinking status.

**Table 4:** Energy intake based on the 4-Day Food Diary according to alcohol consumption.

	Women					Men				
	Alcohol category	mean±SD*	β**	95% CI	P-value	Alcohol category	mean±SD*	β**	95% CI	P-value
Total energy intake, Kcal	None (n = 604)	1544±421	ref			None (n = 252)	2084±600	ref		
	Moderate (n = 252)	1601±439	0.03	−0.01, 0.07	0.136	Moderate (n = 444)	2160±567	0.04	−0.01, 0.08	0.093
	Heavy (n = 48)	1635±438	0.03	−0.05, 0.11	0.472	Heavy (n = 120)	2048±566	−0.01	−0.07, 0.06	0.840
Fat, E%	None (n = 604)	32.4±5.6	ref			None (n = 252)	33.7±5.9	ref		
	Moderate (n = 252)	33.8±5.8	1.24	0.36, 2.11	0.006	Moderate (n = 444)	34.7±5.9	1.05	0.10, 2.00	0.030
	Heavy (n = 48)	34.3±7.0	1.09	−0.66, 2.85	0.222	Heavy (n = 120)	33.8±6.2	−0.15	−1.51, 1.22	0.834
SAFA, E%	None (n = 604)	13.7±3.2	ref			None (n = 252)	14.2±3.7	ref		
	Moderate (n = 252)	14.1±3.0	0.28	−0.20, 0.76	0.246	Moderate (n = 444)	14.5±3.5	0.37	−0.19, 0.93	0.191
	Heavy (n = 48)	14.4±3.4	0.14	−0.84, 1.10	0.782	Heavy (n = 120)	13.9±3.3	−0.35	−1.15, 0.45	0.391
MUFA, E%	None (n = 604)	10.4±2.4	ref			None (n = 252)	10.9±2.3	ref		
	Moderate (n = 252)	11.0±2.4	0.56	0.19, 0.93	0.003	Moderate (n = 444)	11.4±2.4	0.42	0.04, 0.81	0.032
	Heavy (n = 48)	11.3±3.0	0.68	−0.06, 1.42	0.072	Heavy (n = 120)	11.3±2.7	0.26	−0.30, 0.82	0.364
PUFA, E%	None (n = 604)	4.7±1.3	ref			None (n = 252)	4.9±1.4	ref		
	Moderate (n = 252)	5.0±1.5	0.30	0.09, 0.51	0.006	Moderate (n = 444)	5.1±1.4	0.19	−0.04, 0.41	0.100
	Heavy (n = 48)	5.1±1.5	0.37	−0.05, 0.80	0.085	Heavy (n = 120)	4.9±1.4	−0.06	−0.39, 0.26	0.700
Carbohydrates, E%	None (n = 604)	50.0±5.8	ref			None (n = 252)	49.3±6.2	ref		
	Moderate (n = 252)	46.3±6.1	−3.48	−4.38, −2.59	<0.001	Moderate (n = 444)	45.3±6.6	−3.49	−4.51, −2.46	<0.001
	Heavy (n = 48)	42.2±7.3	−6.47	−8.28, −4.67	<0.001	Heavy (n = 120)	40.4±7.1	−7.56	−9.04, −6.08	<0.001

Abbreviations: SAFA = Saturated fatty acids; MUFA = Monounsaturated fatty acids; PUFA = Polyunsaturated fatty acids.

* unadjusted means ± standard deviation.

** beta coefficient adjusted with age, occupation, marital status, smoking, body mass index and leisure time physical activity.

## Discussion

### Food Intake

Alcohol consumption was associated with certain food intake in both men and women. Increasing alcohol consumption was associated with higher fish intake while male heavy drinkers had lower intake of milk than non-drinkers. This is consistent with the study of Ruf et al [19], who found that there were increased intakes observed for animal products such as beef, eggs, fish and low intake of dairy products among participants who consumed high amount of alcohol. Likewise, Männistö and colleagues [20] found that intake of fish and poultry products was associated with increased alcohol drinking while milk and cream intakes reduced with alcohol consumption. Similarly, a Danish study found that alcohol intake (especially wine) was associated with higher intake of fish and also fruits in both men and women [21]. In our study, we also found in crude analyses that moderate drinkers have higher fruit intake than non-drinkers. However, when adjusted for confounders, the association changed direction which means that higher fruit intake in moderate drinkers was explained by other factors. Overall, moderate alcohol consumption has been found to be associated with healthy dietary lifestyles compared with other drinkers or non-drinkers [21], [22], which was not confirmed in our study.

### Energy Intake

Previous studies have found that increased consumption of ethanol, increases total daily energy intake significantly [6], [19]. We found no difference in total energy intake between alcohol consumption groups. However, the participants were instructed to abstain from alcohol consumption during the 4-day Food diary record period, thus the higher BMI among female heavy drinkers may be due to excess energy from their usual alcohol consumption. On the other hand, we did find that the sources of energy were different between moderate/heavy drinkers compared to non-drinkers. Consistent with earlier studies [20], [19], alcohol drinkers tend to get energy from fats while energy from carbohydrate intake was lower than in non-drinkers.

### Nutrient Intake

The lower intake of fibre observed in heavy drinkers corroborate with the findings of Ruf et al [19]. Low consumption of fibre detected among heavy alcohol consumers could lead to harmful health effects if the observed nutritional habits are continued over a long period of time. In a recent prospective study among middle-aged people in several states in United States, dietary fibre intake lowered the risk of mortality from cardiovascular diseases, communicable and pulmonary diseases by 24% to 56% in men and by 34% to 59% in women [23]. A Finnish cohort study by Pietinen et al [24] found that higher intake of fibre may be protective against coronary heart disease especially coronary mortality. Furthermore, previous study conducted in our study sample (the KIHD data) found that daily intake of high folate was associated with decrease in the risk of acute coronary event [15]. This means that heavy drinkers may be in an increased risk of coronary events due to low fibre [24] and folate intakes [15]. We have previously reported that heavy drinkers in the KIHD study have an increased risk of myocardial infarction [25] and increased risk of CVD [26] among men.

Moderate and heavy consumers of alcohol had a significantly higher intake of vitamin D in comparison with non-drinkers. This might be attributed to the higher fish consumption. This finding was consistent with the results of a recent study by Matsui et al [27], who found relatively high levels of vitamin D in male heavy drinkers during their interventional study. Possible explanation may also be a physiological compensation for potential calcium deficiency that arises in heavy alcohol consumers as a result of higher levels of vitamin D. This will eventually lead to maintenance of the calcium homeostasis [28]. However, in our analyses calcium intake was only slightly lower among heavy drinkers compared to non-drinkers.

We found an association between alcohol consumption and BMI as well as energy expenditure based on leisure time physical activity in women but not in men. The lower BMI in the female moderate drinkers compared to non-drinkers partly substantiate the findings by Colditz et al. [29], Barefoot et al. [7] and Ruf et al. [19], in which an inverse relationship between alcohol consumption and BMI was detected. Different form of physical activity may be responsible for these findings [30]. This also confirms our results that female non-drinkers had lower energy expenditure by conditioning leisure time physical activities compared moderate drinkers. However, in contrast to other studies [29], [30], we found that female heavy drinkers had higher BMI and lower physical activity than non and moderate drinkers.

Compared to the situation in 1998/2001 with current state, the unemployment rate was higher and the consumption expenditure was lower in all occupation groups [31]. The consumption expenditure specifically to alcoholic beverages increased among blue and white collar workers and decreased among farmers [32]. Furthermore, alcohol tax was reduced by 33% in 2004, although increased by 10% in 2009 [33]. Overall, the affordability of alcoholic beverages today is higher than at the time of the study. In a companion paper by Fawehinmi et al [34], performed on the baseline population of the KIHD Study; we reported that male heavy drinkers aged 42–60 years had a lower intake of milk, whole grains, fibre, folate, vitamin C, tea and iron than moderate and non-drinkers. Higher intake of legumes, fish and beef was found among heavy drinkers compared to moderate and non-drinkers. These results are similar to our current study conducted 11 years after the baseline.

### Strengths and Limitations

The fairly large sample size, being representative of the population of Eastern Finland is one of the strengths of this research. Another strong point of our study was that we were able to analyze men and women separately and also to adjust the analyses for age, occupational status, marital status, smoking, BMI and physical activity. However, the group of heavy drinking women was relatively small (n = 48) which limits making firm conclusions on this group. Limitations of this study include its cross-sectional nature which does not allow for establishment of causal association. In the future, longitudinal studies are needed to assess causal association between alcohol consumption and dietary patterns. Alcohol consumption was measured by self-report and people tend to under-report their alcohol consumption. It is also possible that there is a lack of interest for chronic alcohol drinkers to fill in the questionnaire, thus affecting the quality of the information obtained and introduction of information bias [35]. The mean age of the participants was 61 years (women) and 62 years (men). The association between alcohol consumption and dietary patterns might be different among younger people who have different drinking habits [36].

### Conclusions and Public Health Implication

The harmful effects of diet rich in fat and low in some essential vitamins in the development of chronic illness are perceived as the leading cause of high mortality in developing and developed countries [37]. In this study, we showed that male participants who were heavy drinkers had a lower intake of several nutrients, such as calcium, iron and fibre compared to non-drinkers. Sufficient intake of essential nutrients is important to prevent chronic diseases such as cardiovascular diseases and osteoporosis which are related to lack of these nutrients. Osteoporosis increases the risk of hip fractures which is a strong predictor of mortality [38]. Cardiovascular diseases (CVD) are key causes of premature deaths, morbidity and disability in nearly all countries [39]. Furthermore, alcohol consumption is a known factor particularly for an increase in CVD risk [40]–[42]. CVDs are the leading causes of deaths in Finland being responsible for 39% of all deaths among men [43]. Numerous CVD risk factors have now been proven to be decreased with eating habits that are in-line with the current guideline recommendations. Many interventional and epidemiological studies have found that a number of dietary patterns and light-to-moderate alcohol consumption is cardio-protective [44]–[46]. Mediterranean diet, which contains alcoholic beverages (typically red wine) add an autonomous cardiovascular benefit to this diet [47]. It has also been reported that light-to-moderate alcohol consumption results into decrease in CVD deaths [48] as well as reduction in the risk of stroke [49]. Menotti and colleagues [44] investigated the food intake habits and 25 year mortality from CHD risk in seven countries. Their findings revealed that intake of vegetables, fish and alcohol was inversely associated with CHD mortality. People on mediterranean diet (MD) (high amount of vegetables, olive oil, alcohol and fish) had lowest mortality from the seven countries included in the study. In elderly people, healthier dietary patterns like MD was associated with lower likelihood of having CVD risk factors [50]. These findings propose that MD has an important effect in protecting CVDs. It is important that health care professionals are aware of the possible nutrient deficiency among middle-aged and older men based on their alcohol consumption levels. More attention should be paid to nutrient intake and safe levels of alcohol consumption for those who drink large amount of alcohol.
